# What is the impact of user affect on motor learning in virtual environments after stroke? A scoping review

**DOI:** 10.1186/s12984-019-0546-4

**Published:** 2019-06-27

**Authors:** Nina Rohrbach, Emily Chicklis, Danielle Elaine Levac

**Affiliations:** 1 Chair of Human Movement Science, Department of Sport and Health Sciences, Technical University of Munich, Munich, Germany; 2 Department of Physical Therapy, Movement & Rehabilitation Science, Northeastern University, Boston, MA USA

**Keywords:** Virtual reality, Stroke, Motor learning, Motivation, Enjoyment, Engagement, Immersion, Presence, Scoping review

## Abstract

**Purpose:**

The purported affective impact of virtual reality (VR) and active video gaming (AVG) systems is a key marketing strategy underlying their use in stroke rehabilitation, yet little is known as to how affective constructs are measured or linked to intervention outcomes. The purpose of this scoping review is to 1) explore how motivation, enjoyment, engagement, immersion and presence are measured or described in VR/AVG interventions for patients with stroke; 2) identify directional relationships between these constructs; and 3) evaluate their impact on motor learning outcomes.

**Methods:**

A literature search was undertaken of VR/AVG interventional studies for adults post-stroke published in Medline, PEDro and CINAHL databases between 2007 and 2017. Following screening, reviewers used an iterative charting framework to extract data about construct measurement and description. A numerical and thematic analytical approach adhered to established scoping review guidelines.

**Results:**

One hundred fifty-five studies were included in the review. Although the majority (89%; *N* = 138) of studies described at least one of the five constructs within their text, construct measurement took place in only 32% (*N* = 50) of studies. The most frequently described construct was motivation (79%, *N* = 123) while the most frequently measured construct was enjoyment (27%, *N* = 42). A summative content analysis of the 50 studies in which a construct was measured revealed that constructs were described either as a rationale for the use of VR/AVGs in rehabilitation (76%, *N* = 38) or as an explanation for intervention results (56%, *N* = 29). 38 (76%) of the studies proposed relational links between two or more constructs and/or between any construct and motor learning. No study used statistical analyses to examine these links.

**Conclusions:**

Results indicate a clear discrepancy between the theoretical importance of affective constructs within VR/AVG interventions and actual construct measurement. Standardized terminology and outcome measures are required to better understand how enjoyment, engagement, motivation, immersion and presence contribute individually or in interaction to VR/AVG intervention effectiveness.

**Electronic supplementary material:**

The online version of this article (10.1186/s12984-019-0546-4) contains supplementary material, which is available to authorized users.

## Introduction

An increasing evidence base supports the use of virtual reality (VR) and active video gaming (AVG) systems to promote motor learning in stroke rehabilitation [1–4]. However, practical and logistical barriers to VR/AVG implementation in clinical sites have been well described [5–7]. To support their use, researchers and developers often emphasize the potential advantages of VR/AVG systems over conventional interventions, including that these technologies may enhance a patient’s affective experience in therapy for the purpose of facilitating recovery [8–11]. Examining the role of affective factors for motor learning is an emerging area of emphasis in rehabilitation [2, 12–15].

VR/AVG use may enhance patients’ motivation to participate in rehabilitation as well as their engagement in therapeutic tasks. Motivation encourages action toward a goal by eliciting and/or sustaining goal-directed behavior [16]. Motivation can be intrinsic (derived from personal curiosity, importance or relevance of the goal) or extrinsic (elicited via external reward) [17]. Engagement is a cognitive and affective quality or experience of a user during an activity [16]. Many characteristics of VR/AVG play can contribute to user motivation and engagement, such as novelty, salient audiovisual graphics, interactivity, feedback, socialization, optimal challenge [14], extrinsic rewards, intrinsic curiosity or desire to improve in the game, goal-oriented tasks, and meaningful play [18].

Motivation and engagement are hypothesized to support motor learning either indirectly, through increased practice dosage leading to increased repetitive practice, or directly, via enhanced dopaminergic mechanisms influencing motor learning processes [15, 16]. Yet evidence is required to support these claims. A logical first step is to understand how these constructs are being measured within VR/AVG intervention studies. Several studies have used practice dosage or intensity as an indicator of motivation or engagement [19–21]. To the authors’ knowledge, few have specifically evaluated the indirect mechanistic pathway by correlating measurement of patient motivation or engagement in VR/AVGs with practice dosage or intensity. While participants in VR/AVG studies report higher motivation as compared to conventional interventions [22–24], conclusions regarding the relationship between motivation and intervention outcomes are limited by lack of consistency and rigour in measurement, including the use of instruments with poor psychometric properties [22, 23].

The body of research exploring the *direct* effects of engagement or motivation on motor learning is still in its infancy. Lohse et al. [16] were the first to evaluate whether a more audiovisually enriched as compared to more sterile version of a novel AVG task contributed to skill acquisition and retention in typically developing young adults, finding that participants who played under the enriching condition had greater generalized learning and complex skill retention. Self-reported engagement (User Engagement Scale; UES) was higher in the enriched group, but the only difference in self-reported motivation was in the Effort subscale of the Intrinsic Motivation Inventory (IMI), where the enriched group reported less effort as compared to the sterile group. The authors did not find a significant correlation between engagement, motivation and retention scores. A follow-up study using electroencephalography did not replicate the finding that the more enriched practice condition enhanced learning, it did show that more engaged learners had increased information processing, as measured by reduced attentional reserve [25].

Enjoyment, defined as ‘the state or process of taking pleasure in something’ [26], has less frequently been the subject of study in motor learning research, but has become popular as a way of describing patient interaction with VR/AVGs. Enjoyment may be hypothesized to be a precursor to both motivation and engagement. Given that the prevailing marketing of VR/AVGs is that they are ‘fun’ and ‘enjoyable’ [1, 3, 14, 27], it is important to evaluate its measurement in the context of other constructs.

Motivation, engagement and enjoyment in VR/AVGs may be influenced by the additional constructs of immersion and presence. Immersion is defined as “the extent to which the VR system succeeds in delivering an environment which refocuses a user’s sensations from the real world to a virtual world” [13, 28]. Immersion is considered as an objective construct referring to how the computational properties of the technology can deliver an illusion of reality through hardware, software, viewing displays and tracking capabilities [29, 30]. A recent systematic review [13] could not conclusively state effect of immersion on user performance. Immersion is distinct from presence, defined as the “psychological product of technological immersion” [31]. Presence is influenced by many factors, including the characteristics of the user, the VR/AVG task, and the VR/AVG system [28]. While presence is thought to be related to enhanced motivation and performance [32], relationships between this and other constructs of interest require exploration. Table 1 outlines definitions of constructs of interest to this scoping review.

**Table 1:** Construct definitions

Construct	Definition	Reference
Motivation	Motivation encourages action toward a goal by eliciting and/or sustaining goal-directed behavior.	[16]
Engagement	Engagement is a cognitive and affective quality or experience of a user during an activity.	[16]
Enjoyment	The state or process of taking pleasure in something.	[26]
Immersion	The extent to which the VR system succeeds in delivering an environment which refocuses a user’s sensations from the real world to a virtual world.	[13, 28]
Presence	The psychological product of technological immersion.	[31]

The purpose of this scoping review is to explore the impact of these affective constructs on motor learning after stroke. This greater understanding will enhance the clinical rationale for VR/AVG use and inform directions for subsequent research. Specifically, our objectives were to:

1. Describe how VR/AVG studies measure or report client enjoyment, motivation, engagement, immersion and presence.
2. Evaluate the extent to which motivation, enjoyment, engagement, immersion, and presence impact motor learning.
3. Propose directional relationships between enjoyment, motivation, engagement, immersion, presence and motor learning.

## Methods

Scoping reviews synthesize knowledge about an exploratory research question to map a field of literature [33]. They are useful methodologies to address questions beyond effectiveness and to describe how a particular subject has been conceptualized or studied [33]. The study is structured according the original methodological framework for conducting scoping reviews [34] and the updated recommendations proposed by Levac et al. [35]. The updated methodological framework consists of six stages: stage 1) Identifying the research question; Stage 2) Searching for relevant studies; 3) Selecting studies; 4) Charting the data; 5) Collating, summarizing, and reporting the results, and 6) Consulting with stakeholders to inform or validate study findings. The consultation stage is optional and was omitted here. The review follows the PRISMA-ScR (Preferred Reporting Items for Systematic reviews and Meta-Analyses extension for Scoping Reviews) reporting guidelines [36].

### Protocol and registration

The protocol was registered with the Open Science Framework on 8 November 2017 (OSF, http://osf.io/3x6y5) after the development of the search strategy and before data extraction and analysis.

#### Stage 1. Identifying the research questions

Our research questions (RQ) were as follows:

- RQ1: How have studies of VR/AVGs in stroke rehabilitation measured or described motivation, enjoyment, engagement, immersion or presence?
- RQ2: What is known as to the extent to which motivation, enjoyment, engagement, immersion and presence impact training outcomes?
- RQ3: What are the proposed relationships between motivation, enjoyment, engagement, immersion, presence and motor learning?

#### Stage 2. Identifying relevant studies

##### Information sources

CINAHL Complete, MEDLINE, and PEDro were searched for articles published from 2007 up to November 2017. This timeline was chosen on the basis of the rapid development in the field following the release of the active video gaming system Nintendo Wii/WiiFit in 2007. The specific search strategy was created by one author (NR) and peer reviewed by another author (DL) with expertise in conducting scoping reviews. A combination of medical sub-headings (MeSH) and key words on “stroke” and “virtual reality”, were adapted as needed for each database and combined using boolean operators.

##### Search

The MEDLINE search strategy is exemplarily presented in Additional file 1: Table S1 (Date of last search: Friday, October 13, 2017 4:16:49 PM).

#### Stage 3. Study selection

##### Eligibility criteria

The Population-Intervention-Comparison-Outcome (PICO) approach was applied to systematically define our eligibility criteria. Inclusion criteria were studies of any design published in the last 10 years in English or German describing rehabilitation interventions using virtual reality (VR) and/or active video games (AVG) including both commercially available systems (i.e. Nintendo Wii console, Sony PlayStation or Xbox Kinect consoles) and custom designed games for stroke rehabilitation (including interfaces such as handheld controllers, gloves, treadmill, etc.) for motor skill improvement in adult patients with stroke. We did not specifically search for studies measuring any of the five constructs. We included studies with any type of control as long as data provided for stroke population were reported separately. Exclusion criteria were robot-based interventions and robot-assisted training (exoskeletons, fixed manipulandum), functional electrical stimulation/ transcranial current stimulation; brain computer interface, electromyography-controlled interventions; outcomes other than motor-based (i.e. energy expenditure, metabolism, cognitive, memory, communication, neglect), and usability/reliability studies, reviews or meta-analyses. Robotic devices, even those that include VR simulations, were excluded due to the additional potential influences on our constructs of interest resulting from the physically assistive nature of these devices.

### Selection of sources of evidence

**Fig. 1:**
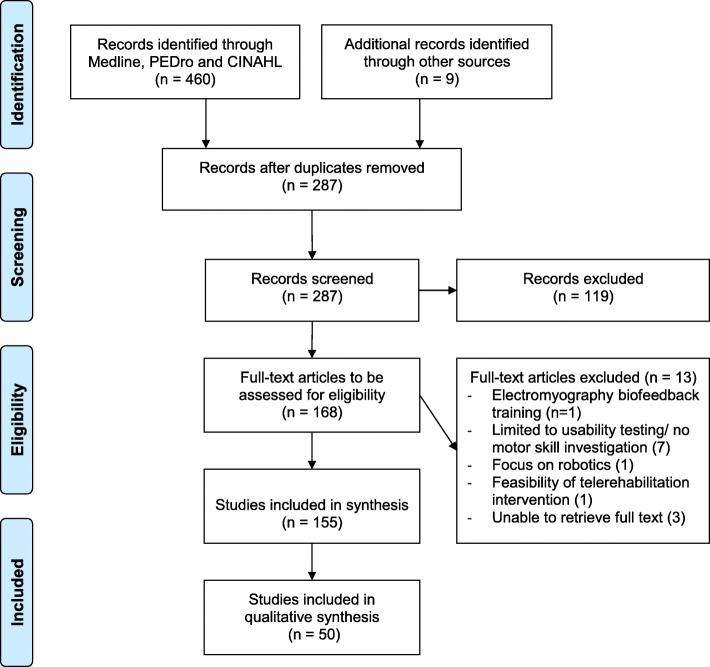
Flow chart depicting the selection process of identified articles

Prior to the formal screening process, a calibration exercise with two reviewers (JZ, NR) on a subset of articles (*n* = 10) was undertaken to pilot the screening questions and eligibility criteria. Based on that calibration exercise, the first selection was made by title and abstract screening of each study by one reviewer (NR). Studies that did not meet the eligibility criteria on the basis of the content of their abstracts were excluded. If required, the full-text versions were obtained to determine whether the studies met our eligibility criteria. In case of uncertainty, another reviewer (DL) blind to the first reviewers’ comments reviewed the study. Any disagreements concerning the inclusion/exclusion were collaboratively discussed until the authors met consensus. Figure 1 outlines the study selection process [37].

#### Stage 4. Charting the data

##### Data charting process

Data from the identified studies were extracted using a charting framework developed a priori by the authors. The charting framework was pilot-tested with a random sample of five articles to check agreement among reviewers. As a result of this process, charting rules were developed to guide a group of five reviewers, including the original two (NR, DL, KP, JZ, MS), who independently charted data from each eligible article. The construct ‘enjoyment’ was added following data extraction from the first 50 papers, as reviewers noted that it was a frequently mentioned construct and should be included in the review; these papers were re-reviewed. The full process required frequent review and discussion within the core research team (NR, EC, and DL) to resolve any uncertainties, and ensure that data extraction was in line with the research questions. All charted data from each reviewer was reviewed by one of the authors (NR). EndNote X8 was used as a reference management software and to avoid multiple reports of the same study. Microsoft Excel was used to manage data within the review team.

##### Data items

The main data extracted were 1) study characteristics (journal name, year of publication, study design, type of VR/AVG technology, study sample, duration of intervention); 2) text in which the author mentioned or described each of the five constructs (motivation, enjoyment [also described as ‘fun’], engagement, immersion, presence); 3) nature of measurement of any of the five constructs (inferential or qualitative), and 4) text related to the authors’ proposed relationships between the constructs.

##### Characteristics and critical appraisal of individual sources of evidence

We did not appraise the methodological quality or risk of bias of the included studies, which is consistent with guidance on scoping review conduct [36].

#### Stage 5. Collating and summarizing the results

##### Synthesis of results

**Fig. 2:**
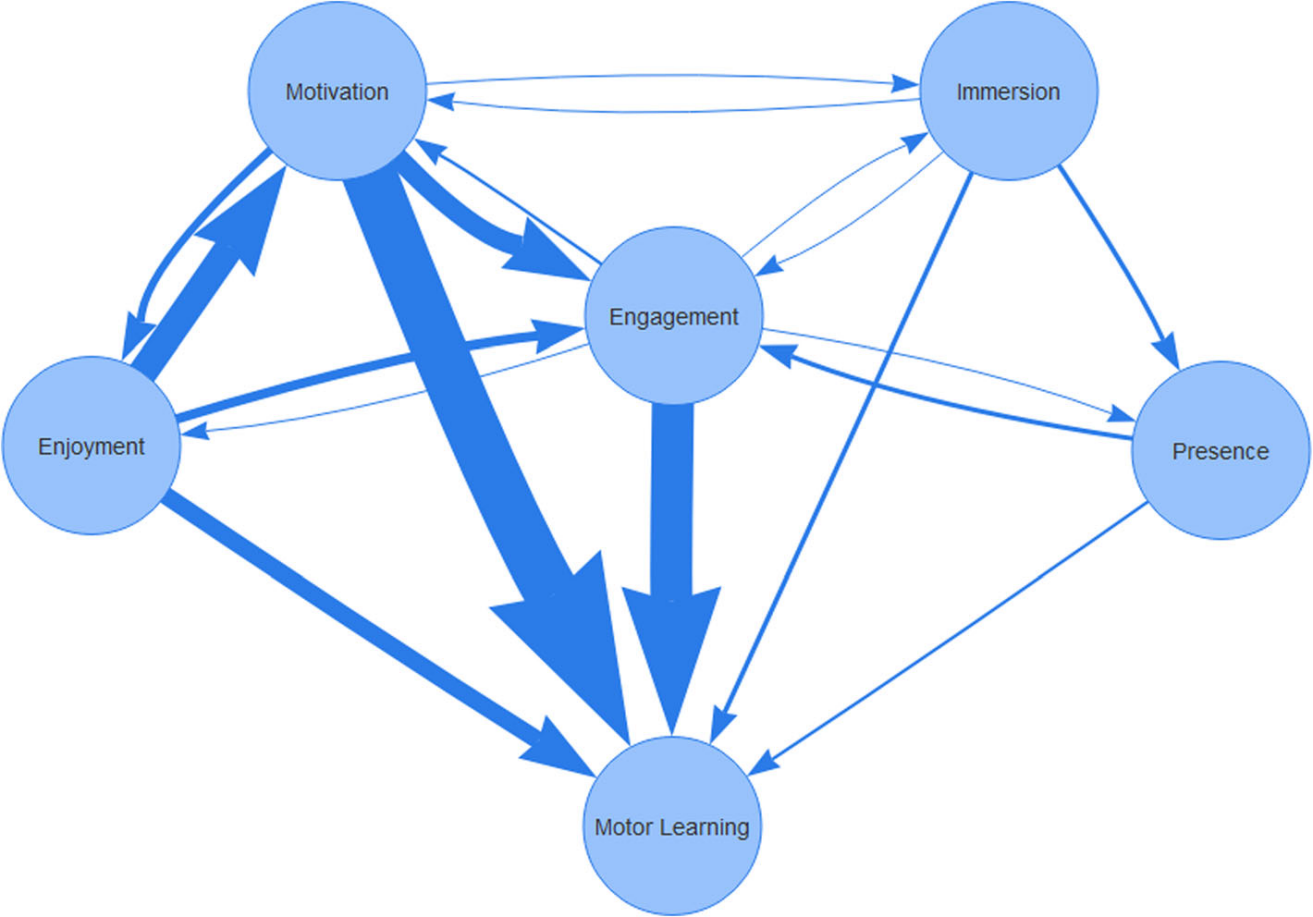
Proposed relationships between the five constructs and motor learning

Numerical analysis (counts, frequencies, proportions) was used to map the studies included in the review in terms of study design, type of VR/AVG system and viewing medium, intervention focus (upper vs lower extremities vs postural control), frequency of mention of constructs, and frequency of measurement of constructs. In a first step, we screened the articles for the constructs of interest (if, and where, the construct was mentioned). In a second step, the studies that mentioned one or more constructs were checked to clarify whether and how the authors measured these constructs (Additional file 2). If measurement was undertaken, the article was included for further analysis (Additional file 3). A numerical summary was used to describe the frequency and type of inferential statistics per construct, and the results of the statistical analyses were summarized. Summative content analysis [38] of the authors’ construct description within the text was undertaken for the subset of studies in which a construct was measured. The goal of summative content analysis is to understand and identify how words are used in context (in this case, we were interested in the specific labels of each of our 5 constructs) [38]. In addition to counting frequencies of use, this approach interprets how words are used and how they relate to each other. Summative content analysis was also used to identify how authors’ described relationships between motivation, engagement, enjoyment, immersion, presence and motor learning in their texts. This analysis resulted in frequency counts of each relationship, which we illustrated in a Fig. 2.

## Results

### Selection of sources of evidence

Figure 1 outlines the study selection process.

#### Overview of the included studies

The articles included in this review employed a range of methodologies: 45.2% were Randomized Controlled Trials (RCTs, *n* = 70), 18.7% were pilot-studies (*n* = 29), 9.7% were pilot-RCTs (*n* = 15), 10.3% used a pre-post design (*n* = 16), 5.8% were case reports/series (*n* = 9), and 10.4% (*n* = 16) applied other designs such as mixed-methods, interviews, non-randomized controlled or crossover trials, overviews of work, case control or descriptive observational studies. Table 2 illustrates that most studies (47%, *n* = 73) involved customized, rehabilitation-specific devices in which tracking/interaction took place indirectly via a controller (e.g., the YouGrabber system) or directly via motion capture (e.g., using the Microsoft Kinect sensor) and visual display of the virtual environment was on a 2D flat-screen monitor, while only 5 % of studies (*n* = 8) investigated any type of stereoscopic glasses or head mounted display. Most (66.45%) of included studies (*n* = 103) focused on upper extremity impairments after stroke while 29.67% (*n* = 46) focused on lower extremities/balance and 3.8% focused on both (*n* = 6). Additional File 2 provides a complete list of the 155 studies included in the review.

**Table 2:** VR classification

	Markerless/Motion Capture		Controller/Peripheral	
	Non-customized	Customized	Non-customized	Customized
Head mounted display	0	3	0	5
Screen/projection	13	35	37	73
Stereoscopic 3D glasses	0	0	0	3

#### RQ1: How have studies of VR/AVGs in stroke rehabilitation described or measured motivation, engagement, enjoyment, presence or immersion?

One hundred fifty-five studies were included in the review. 89% (*n* = 138/155) of studies mentioned at least one of the five constructs within their text, but only 32% (*N* = 50/155) measured a construct using a standardized or non-standardized outcome measure (Table 3).

**Table 3:** Frequency of construct measurement and mention

	Mentioned/described	Measured
Motivation	123/155 (79.35%)	28/155 (18.06%)
Enjoyment/Fun	73/155 (47.09%)	42/155 (27.09%)
Engagement	65/155 (41.93%)	8/155 (5.16%)
Immersion	47/155 (30.32%)	4/155 (2.58%)
Presence	17/155 (10.96%)	6/155 (3.87%)

Table 4 lists the outcome measures used per construct. Examples of standardized measurements include the Intrinsic Motivation Inventory (IMI) and the Presence Questionnaire (PQ). Self-designed questionnaires were applied in 15 studies, where authors mostly used Likert scales or dichotomous yes/no answer formats to evaluate motivation, engagement and enjoyment/fun. For example, Chen et al. [52] designed specific questions to assess motivation and enjoyment that were scored on a 5-point Likert-type scale, with 1 signifying “strongly disagree” and 5 being “strongly agree”. Another example can be found in Schuck et al. [53] who asked questions requiring yes/no answers such as “Was the game fun to play? Did the game increase your motivation to perform your exercise?”, Summative content analysis [38] of the authors’ construct description within the text was undertaken for the 50 studies in which a construct was measured. Additional File 3 provides a complete list of the 50 studies included in summative content analysis.

**Table 4:** Name and frequency of outcome measures used per construct

Construct	Outcome Measurements	Frequency (N)	References
Motivation	IMI	12	[20, 39–49]
	BDI	2	[50, 51]
	Self-designed Questionnaires^a^	6	[48, 52–56]
	Flow-Questionnaire	1	[57]
	Time system was used	1	[21]
	Interviews/Comments/Surveys	10	[21, 42, 50, 58–64]
Enjoyment/Fun	Sub-scale of IMI (Interest/enjoyment)	10	[20, 39–45, 47, 48]
	Self-designed Questionnaires^a^	12	[49, 52–54, 65–72]
	Flow-Questionnaire	1	[57]
	PACES	1	[73]
	SFQ	3	[74–76]
	Interviews/Comments/Surveys	16	[50, 58–62, 64, 66, 68, 77–82]
Engagement	Self-designed Questionnaires^a^	2	[56, 68]
	Interviews/Comments/Surveys	5	[20, 60, 61, 68, 83]
	Diaries (training time and duration)	1	[20]
	PQ (involvement items: 5,6,10,23,32)	1	[19]
	Training time	1	[19]
	Flow-Questionnaire	1	[57]
Immersion	ITQ	1	[78]
	SFQ	2	[76, 84]
	PQ (involvement items: 5,6,10,23,32)	1	[19]
Presence	ITQ	1	[78]
	SFQ	4	[74–76, 84]
	PQ (involvement items: 5,6,10,23,32)	1	[19]

*IMI* Intrinsic Motivation Questionnaire, *BDI* Beck Depression Inventory with four sections: cognitive, emotive, motivational, physiological; ^a^ e.g. VAS/Likert format, *PACES* Physical ACtivity Enjoyment Scale, *PQ* Presence Questionnaire, *ITQ* Immersive Tendencies Questionnaire, *SFQ* Short Feedback Questionnaire

Two themes emerged from the content analysis. In the first theme, represented in 76% of studies (*n* = 38/50), authors described the construct as a rationale for use of VR/AVGs in rehabilitation. In the second theme, represented in 58% of studies (*n* = 29/50), authors used the construct to explain why the VR/AVG intervention was successful. The two themes are described below. Table 5 depicts the quantitative breakdown of both themes for each individual construct.

**Table 5:** Quantitative summary of summative content analysis

	Measured	Theme 1: Construct mentioned as a rationale for use of VR/AVG	Theme 2: Construct mentioned as an explanation for successful intervention
Motivation	28/155	21/28 (75%)	23/28 (82.1%)
Enjoyment/Fun	42/155	25/42 (59.52)	17/42 (40.47)
Engagement	8/155	5/8 (62.5%)	5/8 (62.5%)
Immersion	4/155	4/4 (100%)	0/4 (0%)
Presence	6/155	4/6 (66.7%)	0/6 (0%)

##### Theme 1: Construct described as a rationale for use of VR/AVG

Each of the five constructs was described under this theme. Engagement and motivation were described almost identically. Authors described engagement as a rationale for VR/AVG use because of its potential to influence practice dosage and adherence, greater amounts of which were felt to promote functional improvements [19, 20, 56, 57, 68]. Motivation was also described as a rationale for use of VR/AVG interventions for its potential to increase training intensity, influencing motor learning and neuroplasticity [20, 39, 41–45, 49, 60]. The ability to motivate clients in this way was identified as unique to this treatment method [40, 42, 45, 48]. Rationales presented for VR/AVG use included the potential to engage and motivate users by involving them in game selection [20] or individualization of game features [46], the ability to elicit multiplayer competition or cooperation [39, 44, 48] the provision of individualized challenge [21, 44, 48, 54, 57], and the delivery of feedback [43, 45, 50, 53, 61, 62, 73] or of a rewarding sense of achievement [45, 55]. For example, Subramanian et al. [49] stated that “*Motivation and interactivity of the VE were enhanced by the added visual effects and game score that enabled participants to track success.*”

The potential for VR/AVG use to increase patient enjoyment was described as a strong rationale for use in rehabilitation. Specifically, authors outlined patient enjoyment related to playing games [60, 62, 64, 66, 70–72, 82] which differed from traditional rehabilitation approaches (e.g. [52, 73]. Enjoyment was also seen as essential to the flow experience induced by VR/AVG play [57, 72]. Flow was defined as the *“feeling of complete and energized engagement in an activity, with a high level of enjoyment and fulfillment”* and described as supportive of adherence to VR/AVG-based rehabilitation [72]. Enjoyment was described as facilitating motivation [20, 39–45, 47–49, 68, 74], engagement [57, 68, 78], and training intensity [42, 60, 66, 79] in VR/AVG use. Finally, patient enjoyment due to rewards and feedback provided in VR/AVG games was described as a rationale for their use in clinical practice [57, 66, 78].

Immersion was described as a rationale for VR/AVG use because of its influence on user performance and the fact that it differentiates VR/AVG interventions from conventional rehabilitation [76, 84]. Similarly, authors described presence as an essential component separating the advantages of VR/AVG use over other interventions [75, 76, 78, 84]. All authors interpreted immersion as a subjective characteristic, i.e. defining it as *“the perception of the setting as real”* [76, 84], or *“the feeling of being in the virtual world, rather than looking at it”* [78]. For example, Crosbie et al. [78] stated: *“A person with a positive immersive tendency* [as measured by the ITQ instrument] *may be more likely to be successful in the performance of virtual tasks.”* Authors also described the need to measure side effects associated with immersion to justify the burden of VR/AVG use [19, 78, 84].

##### Theme 2: Construct described as an explanation for successful intervention

Motivation, enjoyment and engagement were the only constructs described under this theme. Engagement and motivation were described as contributing to intervention success by promoting adherence and contributing to a higher training intensity [20, 41, 43, 47, 48, 52, 55, 58] as well as by distracting participants’from therapeutic intent [50, 60]. For example, Lewis et al. [68] state that *“the level of engagement and motivation in performing tasks is posited as factor in determining the success of rehabilitation interventions using VR”*. Another example is Sampson et al. [41] who describe that “*(…) perhaps the main benefit found in this study was that the VR system successfully motivated participants to practice using their affected arms and engage in and enjoy therapy for sustained periods of time.”* Game design features such as individualized challenge levels [21, 42, 47, 48, 51–53, 57, 58, 61, 62], meaningful tasks [20, 52, 53, 57, 58], multiplayer platforms [39, 48], and feedback [42, 43, 46, 48, 49, 51, 52, 58, 61–63] were described as promoting motivation and influencing successful outcomes. For example, Friedmann et al. [43] suggested that “...*sensory-rich visual and auditory feedback motivated high effort levels”*.

Enjoyment achieved through VR/AVG play was described as important to intervention outcomes because it is a critical factor for rehabilitation success [53, 68, 72], lowers stress levels [49] and induces flow [57, 60, 72]. Enjoyment was also described as an explanation for the success of the interventions due to its effects on patient motivation and engagement [20, 45, 52, 64, 73], particularly in patients who otherwise lacked interest or motivation to complete normal exercise regimes [39, 58]. Gorsic et al. [48] stated enjoyment led to effort, stating that *“participants enjoyed competitive exercises more than exercising alone, and that this also increased self-reported effort put into the exercise.”* Schuck et al. [53] referred their intervention success to previous literature *“that implicated the importance of fun, motivation, and engagement as critical factors for success in rehabilitation*.”

#### RQ2: What do we know about the extent to which motivation, engagement, presence, enjoyment and level of immersion impact training outcomes?

While 74% of studies (*n* = 37) in which a construct was measured reported only descriptive statistics or qualitative summaries, 26% (*n* = 13) used inferential statistical analysis to evaluate hypotheses related to a construct in different arms of the intervention, e.g. to compare differences in motivation or enjoyment between two studied practice conditions [40, 42, 43, 45, 47, 49, 52, 65, 71, 73]. None of the studies used statistical inference to link any of the five constructs to motor learning outcomes.

#### RQ3: What are the proposed relationships between motivation, engagement, enjoyment, immersion, and presence and motor learning?

Summative content analysis was used to explore authors’ interpretations of construct relationships in their texts. Figure 2 illustrates the frequency and direction of identified relationships.

The most frequently described relationship was motivation leading to motor learning (*N* = 24). For example, Hale et al. [80] state: “*One of the rationales for computer-based rehabilitation is the use of the motivational aspects of the technology to stimulate people to practice repetitive movement to facilitate neuroplasticity and enhance functional movement.”* The second most frequently reported relationship was the influence of patient enjoyment on motivation (*N* = 15). Enjoyment is seen as “*a key factor for increasing motivation*” [74]. However, authors measured this construct using an instrument designed to assess motivation: the Intrinsic Motivation Inventory (IMI). For instance, Lloréns et al. [47] conclude: *“In terms of motivation, the results of the IMI showed that most of the participants found the system enjoyable (...)”.*

The combination of two constructs was suggested to influence a third construct. For example, Kottink et al. [45] suggest that the combination of fun and motivation together lead to engagement, stating: *“The application of videogames in rehabilitation (rehab games) can be regarded as a specific form of VR training, in which the fun and motivational elements of the exercises are emphasized to engage people during their activity.”* Multidimensional relationships, in which constructs are chained, are listed in Table 6.

**Table 6:** Construct relationships proposed by authors

Source	Destination 1	Destination 2	Destination 3	Frequency	References
Motivation	Motor Learning			24	[19, 20, 39, 42–45, 48–51, 53, 55, 57, 58, 60, 66–68, 71, 74, 76, 80, 84]
Motivation	Enjoyment			3	[41, 52, 61]
Motivation	Enjoyment	Motor Learning		2	[52, 61]
Motivation	Engagement			12	[19, 20, 41, 45, 46, 51, 57, 61, 64, 73, 79, 81]
Motivation	Engagement	Motor Learning		5	[20, 46, 57, 73, 79]
Motivation	Immersion	Motor Learning		1	[50]
Enjoyment	Motor Learning			6	[52, 53, 61, 68, 78, 80]
Enjoyment	Motivation			15	[20, 39–42, 44, 47, 49, 51, 62, 64, 66, 68, 73, 74]
Enjoyment	Motivation	Motor Learning		5	[42, 44, 66, 68, 74]
Enjoyment	Motivation	Engagement		4	[20, 51, 64, 73]
Enjoyment	Motivation	Engagement	Motor Learning	2	[20, 73]
Enjoyment	Engagement			6	[45, 49, 57, 68, 72, 78]
Enjoyment	Engagement	Motor Learning		3	[49, 68, 78]
Engagement	Motor Learning			13	[19, 20, 40, 46, 49, 50, 53, 57, 68, 73, 78, 79]
Engagement	Motivation			2	[57, 71]
Engagement	Motivation	Motor Learning		1	[71]
Engagement	Enjoyment			1	[60]
Engagement	Immersion	Motor Learning		1	[50]
Engagement	Presence			1	[40]
Immersion	Motor Learning			2	[50, 78]
Immersion	Motivation			1	[60]
Immersion	Engagement			1	[40]
Immersion	Presence	Engagement		2	[19, 50]
Immersion	Presence	Engagement	Motor Learning	1	[19]
Immersion	Presence	Motor Learning		1	[45]
Presence	Engagement	Motor Learning		1	[46]

For example, Flynn et al. [50] state that engagement and motivation lead to immersion and this impacts therapeutic outcomes: “*Moreover, the virtual environment (VE) provides an engaging and motivating framework for feedback allowing the participant to become immersed in the virtual world and to experience the emotional sense of “winning” in a particular game*.” Turkbey et al. [64] suggest that enjoyment leads to motivation, which leads to engagement: “*It should be remembered that patients’ enjoyment and belief in benefits of a treatment may improve engagement in a therapy and intensity of training as a reflection of increased motivation.”* Finally*,* Hung et al. [73] also describe this relationship: *“One of the most important successes of the Wii Fit training may lie in the pleasure component, which motivates subjects to engage more fully in the program*.”

## Discussion

This scoping review explored how motivation, enjoyment, engagement, immersion and presence were described or measured in VR/AVG studies in stroke rehabilitation. We also sought to identify potential links between these constructs and motor learning outcomes. Although the majority of studies mentioned at least one of the five constructs within their text, construct measurement took place in only 1/3 of studies. Multiple relational links between two or more constructs or between any construct and motor learning were described, though statistical analyses were not used to examine these links.

The emphasis by authors on enjoyment was a surprising finding of this review. Enjoyment was described as important because it underlies both engagement and motivation, and because it is central to essential game design principles of VR/AVG games. However, although it was the most frequently measured construct, it is important to note that measurement of this construct was undertaken with the use of instruments designed for other purposes. This included using instruments measuring flow or intrinsic motivation [85, 86] or self-designed subjective questionnaires lacking psychometric properties [72]. Hung et al. [73] were the only ones to use an enjoyment-specific scale (PACES), although its psychometric properties have not yet been validated in the stroke population or for exercise modalities other than sports [87]. Given that authors appear to consider this construct foundational both to the affective impact of VR/AVGs and to the mechanics of game design, it will be important to achieve consensus on optimal measurement.

A second important finding of the review was the inconsistency with which constructs were mentioned, described, defined and measured in these studies, and the fact that despite lack of tests of statistical inference or even measurement, authors stated assumptions or conclusions about constructs as fact. For example, Shin et al. [57] conclude that their device “*encouraged the patient’s skill development, improved immersion, and motivated further rehabilitation by providing meaningful play, optimal challenge, and a flow experience*” while acknowledging that they did not measure motivation. In addition, definitions did not consistently align with our a-priori understanding of the terms, and were often vague and interchangeable. Indeed, these terms are differentially operationalized and defined in various fields (e.g. psychology, sports medicine, rehabilitation). This issue of ill-defined terminology was identified by some authors [20]. For example, immersion was often described as a synonym for presence, as follows: “*This allows users to experience a high degree of immersion; they feel as if they are in the virtual world, rather than looking at it.”* [78] Presence was also described as an indicator of subjective immersion, for example in [19]: *“(...) presence is a subjective measure used in VR studies to quantify how immersed a user is in a VE.”* Also problematic is the fact that authors use a single instrument to measure several different constructs. For example, immersion was measured using the Presence Questionnaire, the same instrument as that used to measure presence, which was also used to measure what authors’ labelled as engagement [19]. Overall, the inconsistent and varying use of terms, as well as the use of single instruments to quantify different constructs presents a challenge for readers and should be addressed through the development of consistent terminology and a consensus on optimal outcome measures [20].

Among the studies in which a construct was measured, 44% of studies (*N* = 22) used validated instruments (e.g. IMI, IM-TEQ, PQ, ITQ, TSFQ, PACES and SFQ), however, most measures were not verified yet for the targeted purpose (e.g., the PACES), or population (e.g. PQ). Most used either indirect tools (e.g. taking training time or practice duration as a measure of motivation and engagement, *N* = 3), study-specific subjective questionnaires with untested psychometric properties (*N* = 15), or exclusively qualitative assessments (e.g. interviews or comments, *N* = 12) with varying rigour in data analysis (Table 4). Tatla et al. [22, 23] also found a lack of valid instruments used to measure motivation in pediatric interventions for children with cerebral palsy and acquired brain injury. As such, consensus is clearly required on instruments in order to align the field and facilitate interpretation and the advancement of knowledge. Existing instruments could be adapted and validated for use in VR/AVG interventions and with specific target populations. For example, Gil-Gómez et al. [88] have proposed the SEQ (Suitability Evaluation Questionnaire) that is based on the SFQ (Short Feedback Questionnaire) but has been updated to cover specific VR-related items.The use of direct or indirect objective measures of motivation, enjoyment, engagement is an option to overcome challenges of subjective self-report. Indirect measures include recording time spent interacting with the VR/AVG game (as undertaken by [19–21], counting the frequency of repetitions, or measuring the intensity of physical activity (for example, using EMG measurement, as in Zimmerli et al. who considered physical activity intensity as an indicator of engagement in VR/AVG interventions) [89]. Clearly, this indirect approach is not without limitations, as there will always be a multitude of influences besides affective state on adherence, dosage and intensity (for example, the expectation of external rewards, or the pressure to maintain a strict treatment schedule). As such, more direct objective measures are also warranted [20]. Examples include electroencephalography, including use of event related potentials to evaluate attentional demand [25], spectra analysis for indicators of engagement, or other measures such as galvanic skin response, heart rate variability or functional near-infrared spectroscopy [90]. The use of such objective measures may elucidate the neurophysiological processes by which affective state influences motor learning [25].

Perez-Marcos [91] suggests that authors should distinguish between VR hardware and software to evaluate user experiences. Specifically, authors should be more specific about describing the components of their VR/AVG interventions to differentiate between systems, the games themselves, and the resulting user experience [91]. Results of our review indicate that game mechanics such as rewards, feedback, challenge, choice/interactivity, clear goals, and socialization [14] were frequently lauded for their influence on motivation, engagement and enjoyment. These game design features are different from the features of the VR system that is delivering the intervention, and can likely be delivered across different platforms. Interestingly, authors did not link these game design features to immersion or presence, indicating that these constructs are more aligned with the game context than with the viewing medium or interaction modality. Further unpacking the ‘active ingredients’ of VR/AVG interventions, and how they may be attached to game characteristics as opposed to hardware components, is a key area for future research [92, 93].

Results of the review illustrate the discrepancy between the frequency of construct description or actual measurement. One potential explanation is that these constructs are universally accepted as inherent to VR/AVG interventions, and as such, researchers are not compelled to measure them. No conclusions can be made about the potential impact of motivation, enjoyment, engagement, degree of immersion and level of presence on the motor improvements achieved in a VE. We recommend including these analyses in future work, where power analyses permit. Such calculations should be facilitated as the field continues to grow and study designs move beyond the feasibility and pilot study stage in which authors’ focus on demonstrating an effect or differentiating the intervention from traditional care.

### Limitations

This scoping review had several limitations. We identified studies in which the apparent goal of VR/AVG interventions was motor skill improvement; however, the assumption of motor learning as an intervention goal was our own. We used summative content analysis to analyze article text, but did not record nor assign speculative or other intent to authors’ words. As such, and particularly since no inferential statistics were performed in the original articles to support identified relationships, we can assign no weight to relational links identified in this review. While our literature search included the three main rehabilitation-specific databases, literature may have been missed from other databases. In particular, we did not search the IEEE Xplore database, which may have led to more studies on immersion and presence, though perhaps not in a rehabilitation context. In keeping with scoping review conduct recommendations, we did not undertake a quality appraisal of the included studies.

The construct of ‘Flow’ was mentioned in relation to motivation, enjoyment, engagement, and immersion, e.g. by stating that *“flow experience results from a combination of intrinsic motivation and complete immersion in the intervention”* [57] and flow was often described as an indicator of engagement [72, 94]. As such, the omission of flow as a construct relevant to affective state in VR/AVG interventions is a scoping review limitation. Finally, we did not differentiate our analyses between non-customized and customized rehabilitation-specific VR/AVG systems. Non-customized systems are less expensive and accessible, may be easier to use and are most frequently used in clinical practice [5]. Differentiating between these types of VR/AVGs may have helped to elicit any potential differences in the constructs that may be due to potentially more impactful game design principles (such as more abundant audiovisual feedback, or more explicit competition) of commercially-available games as compared to rehabilitation-specific games.

### Next steps for research

Results of this scoping review indicate the need for greater consensus on definitions and terminology. Given the lack of psychometrically-valid outcome measures, integrating greater use of objective measures is essential. Researchers should include hypotheses as to how these constructs influence motor learning. High quality mixed methods research designs may be useful when appropriately conducted using a rigorous framework for design and interpretation [95], as a qualitative component can help to further elucidate what specifically participants found motivating or engaging, and can be used as a complement to explore the validity of self-report quantitative measures or objective measures. Finally, measuring sustainability and changes in these constructs over time can inform decision-making protocols for clinicians to better adjust VR/AVG intervention parameters to sustain motivation and engagement [23].

Greater understanding of the impact of affective state on learning will inform the design of VR interventions that can better exploit attributes found to promote motivation and engagement. Researchers can conduct experiments in VR to inform directions for development of VR-based therapeutic tasks, but they could also provide knowledge to inform conventional rehabilitation by providing greater awareness of the potential importance of affective state for learning. In addition, because VR experimental paradigms can better isolate or manipulate a single task presentation factor over others as compared to experiments in physical environments, this can support understanding of which specific factors enhance motivation and engagement for different types (e.g. ages, interests, cognitive abilities) of users. This can also provide more evidence for why therapists could consider using VR over traditional interventions as well as provide information for how to design conventional interventions that take advantage of these same attributes.

## Conclusions

To accompany the increasing evidence of VR/AVG effectiveness in stroke rehabilitation, it is important to better understand factors that may differentiate certain systems or modulate effectiveness in clients with differing characteristics. The growing emphasis on the role of affective factors in motor learning combined with our findings that many researchers use these constructs as a rationale for VR/AVG use highlight the need to better understand and measure whether affective state differentiates VR/AVG use from traditional interventions and whether it contributes to intervention outcomes. This body of literature currently demonstrates a discrepancy between description and measurement, one that might be explained by the early stage of the literature and the current feasibility-oriented research methodologies. Results of the review provide suggestions for researchers interested in measuring these constructs and emphasize the need for consensus on terminology and outcome measures. Finally, the results point to the need to better understand, through improved measurement and inferential analyses, the potential impact of affective constructs and technical level of immersion on outcomes achieved through practice in VR environments.

## Additional files

Additional file 1: **Table S1.** MEDLINE search strategy. (PDF 106 kb)

Additional file 2: List of studies included in synthesis (*n* = 155). (XLSX 31 kb)

Additional file 3: List of studies included in summative content analysis (*n* = 50). (XLSX 63 kb)

## Data Availability

The datasets used and analysed during the current study are available from the corresponding author on reasonable request.

## Abbreviations

AVG: Active Video Game
BDI: Beck Depression Inventory
IMI: Intrinsic Motivation Inventory
ITQ: Immersive Tendencies Questionnaire
OSF: Open Science Framework
PACES: Physical ACtivity Enjoyment Scale
PICO: Population-Intervention-Comparison-Outcome
PQ: Presence Questionnaire
PRISMA: Preferred Reporting Items for Systematic reviews and Meta-Analyses
RCT: Randomized Controlled Trial
RQ: Research Question
ScR: Scoping Review
SFQ: Short Feedback Questionnaire
UES: User Engagement Scale
VAS: Visual Analogue Scale
VR: Virtual Reality
