# Supplementary material for: What is the impact of user affect on motor learning in virtual environments after stroke? A scoping review
Source: J Neuroeng Rehabil. 2019 Jun 27;16:79. doi: 10.1186/s12984-019-0546-4 (PMC6598261; doi:10.1186/s12984-019-0546-4)
Supplement: Supplementary file 1 — Table S1. MEDLINE search strategy. (PDF 106 kb) [file 12984_2019_546_MOESM1_ESM.pdf]

MEDLINE search strategy
(Date of last search: Friday, October 13, 2017 4:16:49 PM).

#	Query	Limiters/ Expanders	Results
S4	S2 AND S3	Limiters - Age Related: All Adult: 19+ years Search modes - Boolean/Phrase	220
S3	((MH "Stroke") OR "stroke" OR (MH "Stroke Rehabilitation") OR (MH "Cerebrovascular Disorders") OR "poststroke" OR "cerebrovascular disorder") NOT (((MH "Cognition") OR (MH "Cognition Disorders") OR (MH "Cognitive Aging") OR (MH "Cognitive Dysfunction") OR (MH "Perceptual Disorders") OR "neglect" OR (MH "Anxiety") OR "anxiety" OR (MH "Anxiety Disorders")))	Search modes - Boolean/Phrase	268.176
S2	S1 NOT ((MH "Electric Stimulation") OR "functional electrical stimulation" OR "fmri" OR "transcranial magnetic stimulation" OR (MH "Transcutaneous Electric Nerve Stimulation") OR (MH "Transcranial Direct Current Stimulation") OR (MH "Deep Brain Stimulation")) NOT ((MH "Robotics") OR (MH "Exoskeleton Device") OR (MH "Robotic Surgical Procedures") OR robots OR robot assisted therapy OR robot* OR robot assisted training OR exoskeleton*)	Search modes - Boolean/Phrase	12.076
S1	((MH "Virtual Reality Exposure Therapy") OR "virtual reality" OR "augmented reality" OR "mixed reality" OR "virtual rehabilitation" OR "virtual therapy" OR "virtual environment" OR "virtual training") OR (((MH "Video Games") OR (MH "Games, Recreational")) OR “active video gam*” OR "interactive video gam*" OR "serious gam*" OR "exergam*" OR "xbox kinect" OR "sony play*" OR "nintendo wii*")	Search modes - Boolean/Phrase	13.248
